# MiR‐223‐3p inhibits rTp17‐induced inflammasome activation and pyroptosis by targeting NLRP3

**DOI:** 10.1111/jcmm.16061

**Published:** 2020-11-03

**Authors:** Fu‐quan Long, Cai‐xia Kou, Ke Li, Juan Wu, Qian‐qiu Wang

**Affiliations:** ^1^ Department of STD Shanghai Skin Disease Hospital Tongji University School of Medicine Shanghai China; ^2^ Institute of Dermatology Chinese Academy of Medical Sciences and Peking Union Medical College Nanjing China

**Keywords:** inflammasome, microRNA, miR‐223‐3p, NLRP3, syphilis, *Treponema pallidum*

## Abstract

The incidence of syphilis caused by *Treponema pallidum* subsp *pallidum* (*T pallidum*) infection is accompanied by inflammatory injuries of vascular endothelial cells. Studies have revealed that *T pallidum* infection could induce inflammasome activation and pyroptosis in macrophages. MicroRNA‐223‐3p (miR‐223‐3p) was reported to be a negative regulator in inflammatory diseases. The present study aimed to explore whether miR‐223‐3p regulates *T pallidum‐*induced inflammasome activation and pyroptosis in vascular endothelial cells, and determine the mechanisms which underlie this process. MiR‐223‐3p levels in syphilis and control samples were determined. The biological function of miR‐223‐3p in the NLRP3 inflammasome and pyroptosis was evaluated in *T pallidum‐*infected human umbilical vein endothelial cells (HUVECs). We observed a dramatic decrease in miR‐223‐3p levels in syphilis patients (n = 20) when compared to healthy controls (n = 20). Moreover, miR‐223‐3p showed a notable inhibitory effect on recombinant Tp17 (rTP17)‐induced caspase‐1 activation, resulting in decrease in IL‐1β production and pyroptosis, which was accompanied by the release of lactate dehydrogenase (LDH) in HUVECs. Additionally, the dual‐luciferase assay confirmed that NLRP3 is a direct target of miR‐223‐3p. Moreover, NLRP3 overexpression or knockdown largely blocked the effects of miR‐223‐3p on *T pallidum*‐induced inflammasome activation and pyroptosis in HUVECs. Most importantly, a notable negative correlation was observed between miR‐223‐3p and NLRP3, caspase‐1, and IL‐1β, respectively, in the serum of syphilis patients and healthy controls. Taken together, our results reveal that miR‐223‐3p targets NLRP3 to suppress inflammasome activation and pyroptosis in *T pallidum*‐infected endothelial cells, implying that miR‐223‐3p could be a potential target for syphilis patients.

## 1 INTRODUCTION

Syphilis is a multistage and chronic disease caused by *Treponema pallidum* subsp *pallidum* (*T pallidum*), which can involve multiple systems and is associated with significant morbidity. Globally, the incidence of syphilis infection is rising dramatically.^1^, ^2^ The inflammatory processes induced by syphilis within infected tissues result in the development of lesions. Moreover, the up‐regulated expression or activation of NOD‐like receptor (NLR), pyrin domain‐containing (NLRP3) inflammasome, and the subsequent induction of pyroptosis result in excessive inflammation, which is responsible for the pathogenesis of various diseases.^3^, ^4^ Recently, studies have revealed that inflammasome responses are also involved in the development of syphilis‐associated tissue inflammation.^5^, ^6^

Several factors, including bacteria, viruses, fungi, components of dying cells and crystal particles, can activate the NLRP3 inflammasome, which leads to caspase‐1‐dependent maturation of pro‐interleukin(pro‐IL)‐1β and pro‐IL‐18. Eventually, NLRP3 inflammasome activation results in the induction of a cell death mechanism known as pyroptosis, via the cleavage of gasdermin D (GSDMD).^7^, ^8^, ^9^ Recently, the outer membrane protein Tp92 of *T pallidum* was shown to have a potential pathogenic role by inducing a proinflammatory response in macrophages and human microvascular endothelial cells (HMEC)‐1,^10^ as well as inducing caspase‐1‐dependent pyroptosis of mononuclear cells.^11^ Moreover, Li‐Rong Lin *et al* revealed that *T pallidum* infection induced proinflammatory cytokine IL‐1β secretion, which was accompanied by NLRP3 inflammasome activation,^6^ suggesting the importance of the inflammasome and pyroptosis in *T pallidum*‐induced pathology. Therefore, investigation of the factors that regulate NLRP3 inflammasome activation and pyroptosis may lead to the development of effective therapy strategies for syphilis patients.

MicroRNAs (miRNAs) are a highly conserved group of small non‐protein‐coding RNAs that act as post‐transcriptional regulators of gene expression. Currently, there is increasing evidence, which demonstrates that miRNAs are involved in the regulation of many biological processes, including metabolism, inflammation and cancer.^12^, ^13^ Tp17 is a membrane immunogen of *T pallidum,* and our previous work has revealed that miR‐216a‐5p suppresses the recombinant Tp17 (rTP17)‐induced inflammatory response in human umbilical vein endothelial cells (HUVECs) by targeting Toll‐like receptor 4 (TLR4).^14^, ^15^ In addition, another study also reported that *T pallidum* membrane proteins may induce endothelial cell dysfunction and, consequently, affect the progress of the vessel inflammatory response.^10^, ^16^ Thus, we pondered whether there any other miRNAs involved in syphilis pathology. MiR‐223‐3p was previously reported to be up‐regulated in tumour tissues and involved in regulating cell growth and apoptosis via the F‐box/WD repeat‐containing protein 7 (FBXW7), PR domain zinc finger protein 1 (PRDM1), Septin‐6 (SEPT6), p53 and others.^17^, ^18^ In recent years, NLRP3 has also been reported to be a direct target of miR‐223‐3p.^19^ However, the role and mechanism of action of miR‐223‐3p in syphilis remain unknown. Here, we aimed to investigate the effect of miR‐223‐3p in *T pallidum*‐mediated inflammasome activation and pyroptosis in HUVECs. We have found that miR‐223‐3p induced a strong inhibition of *T pallidum*‐induced caspase‐1 activation and IL‐1β production, which indicates that it might be a potential therapeutic target for syphilis.

## 2 MATERIALS AND METHODS

### 2.1 Patients and clinical characteristics

Twenty patients with syphilis (early stage, untreated) were recruited from the Shanghai Skin Disease Hospital, and twenty health individuals were recruited from the Healthcare Center in this hospital (registration ethical approval number: SSDH‐IEC‐SG‐029‐2.1‐2019.16). Syphilis was diagnosed according to the Centers for Disease Control and Prevention’ Sexually Transmitted Diseases Treatment Guidelines.^20^ Blood samples were collected from syphilis patients on the day after diagnosis; the results of blood tests are shown in Table 1. The study was approved by the ethical committee of the Shanghai Skin Disease Hospital, and all patients included in the study provided informed consent.

**TABLE 1:** Information of clinical blood samples for syphilis test

Group	Male/Female	RPR (‐)	RPR1:1	RPR1:2	RPR1:4	RPR1:8	RPR1:16	RPR1:32	RPR ≥ 1:64	TPPA (+)
Healthy controls	13/7	20	0	0	0	0	0	0	0	0
Primary syphilis	6/2	0	2	4	2	0	0	0	0	8
Secondary syphilis	8/4	0	0	0	0	2	2	4	4	12

Abbreviations: RPR, Rapid plasma regain test; TPPA, *Treponemal pallidum* particle agglutination assay.

### 2.2 Cell cultures and materials

HUVECs were purchased from the BeNa Culture Collection and cultured in minimum essential medium (Invitrogen) supplemented with 10% foetal bovine serum (FBS), 100 U/mL penicillin and 0.1 mg/mL streptomycin. The cells, at a density of 10^4^ viable cells/cm^2^, were seeded and then later used for experiments after 48 hours of culturing. The cells at passage 3 were used for this study, with three replicates for each condition.

The *T* *pallidum* Nichols strain was propagated in rabbits as previously described.^16^

### 2.3 Plasmids

The human NLRP3 coding region was cloned into a pCDNA3.0 vector. The 3ʹ‐UTR of the NLRP3 mRNA containing the putative binding site of miR‐223‐3p was amplified and cloned into the luciferase reporter psiCHECK2 vector (Promega) to construct the wild‐type NLRP3‐3ʹ‐UTR^WT^ plasmid. Moreover, the mutant 3ʹ‐UTR of NLRP3 (NLRP3‐3ʹ‐UTR^Mut^) with the seed region for the luciferase reporter was obtained using a KOD Site‐Mutagenesis Kit (Toyobo, Japan). The sequences of the siRNA, miRNA inhibitor and microRNA mimics or inhibitor used in this study are as follows: miR‐223‐3p mimic, sense: 5′‐UGUCAGUUUGUCAAAUACCCCA‐3′, antisense: 5′‐GGGUAUUUGACAAACUGACAUU‐3′; miRNA mimic control, sense: 5′ ‐UUCUUCGAACGUGUCACGUTT‐3′, antisense: 5′‐GGGUAUUUGACAAACUGACAUU‐3′; miR‐223‐3p inhibitor, sense: 5′‐UGGGGUAUUUGACAAACUGACA‐3′, miRNA inhibitor control, antisense: 5′‐CAGUACUUUUGUGUAGUACAA‐3′.

### 2.4 ELISA assay

HUVECs, at a density of 5 × 10^3^ viable cells/cm^2^, were seeded in 96‐well plates. The secretion level of inflammatory cytokine IL‐1β in the tissue or cell supernatant was measured using an enzyme‐linked immunosorbent assay (ELISA) kit (Invitrogen) according to the manufacturer's instructions. Both standards and samples were analysed in triplicate. The optical density (OD) at 450 nm was calculated by subtracting the background, and standard curves were plotted.

### 2.5 Lactate dehydrogenase (LDH) assay

LDH release was quantified from the supernatants using the CytoTox 96 Non‐Radioactive Cytotoxicity Assay (Promega) according to the manufacturer's instructions. Briefly, HUVECs at a density of 5 × 10^3^ viable cells/cm^2^ were seeded in a 96‐well plate and treated with various concentrations of rTp17 for different time periods. Subsequently, each sample aliquot was incubated with the CytoTox 96 Reagent and then with the stop solution. The percentage of LDH release was calculated using the following equation:

LDH release (%)  =  (experimental LDH release ‐ spontaneous LDH release)/maximum LDH release.

### 2.6 Dual‐luciferase reporter assay

HUVECs, at a density of 1 × 10^5^ viable cells/cm^2^, were seeded in 24‐well plates and co‐transfected with the luciferase reporter NLRP3‐3′‐UTR^WT^ or NLRP3‐3′‐UTR^Mut^ and the negative control or miR‐223‐3p mimics (100 nmol/L) using Lipofectamine 2000 (Invitrogen). The cells were harvested at 48 hours after transfection and the firefly and Renilla luciferase activities were detected using the Dual‐Luciferase Reporter Assay System (Promega). The results are presented as the ratio between Renilla and firefly luciferase activity.

### 2.7 Western blotting

HUVECs, at a density of 4 × 10^5^ viable cells/cm^2^, were lysed in ice‐cold RIPA lysis buffer (Beyotime Institute of Biotechnology). Protein concentrations were measured using the BCA protein assay (Beyotime Institute of Biotechnology), according to the manufacturer's instructions. Equal amounts of proteins from each sample were separated via sodium dodecyl sulphate‐polyacrylamide gel electrophoresis (SDS–PAGE) and then transferred onto PVDF membranes. The membranes were then probed with primary antibodies against caspase‐1 p10 (Santa Cruz, USA), NLRP3 (Santa Cruz, USA), IL‐1β (Santa Cruz, USA) or GAPDH (Santa Cruz, USA) overnight at 4°C, followed by incubation with a horseradish peroxidase–conjugated anti‐immunoglobin antibody for 1 hour. The protein bands were visualized using an enhanced chemiluminescent (ECL) HRP substrate (Millipore) and the intensity of the bands was quantified using the Image‐Pro Plus 5.1 Image Analysis Software.

### 2.8 Real‐time reverse transcription‐PCR

Total cellular RNA from human plasma and HUVECs was isolated using the TRIzol reagent (Ambion), according to the manufacturer's instructions. Then, 1 μg of total RNA was reverse‐transcribed into cDNA using a Reverse Transcription Kit (Takara), followed by amplification of the cDNA using the SYBR Green Master Mix (Takara). We analysed three replicates for each sample, on a CFX‐96 real‐time PCR detection system (Bio‐Rad). The PCR reaction conditions were as follows: pre‐denaturation at 95°C for 30 seconds, followed by 40 cycles of 5 seconds at 95°C and 34 seconds at 60°C. The relative expression of the target genes was evaluated using the 2^‐∆∆Ct^ method and normalized to the expression of U6. The sequences of primers used are as follows: miR‐223‐3p, F: 5′‐CGCUAUCUUUCUAUUAACUGACCAUAA‐3′, R: 5′‐CGCUAUCUUUCUAUUAUGACUCCAUAA‐3′; U6, F: 5′‐GTGCTCGCTTCGGCAGCACATATAC‐3′, R: 5′‐AAAAATATGGAACGCTTCACGAATTTG‐3′; GAPDH, F: 5′‐CATGAGAAGTATGACAACAGCCT‐3′, R: 5′‐AGTCCTTCCACGATACCAAAGT‐3′; Caspase 1, F: 5′‐TTTCCGCAAGGTTCGATTTTCA‐3′, R: 5′‐GGCATCTGCGCTCTACCATC‐3′; IL1β, F: 5′‐ATGATGGCTTATTACAGTGGCAA‐3′, R: 5′‐GTCGGAGATTCGTAGCTGGA‐3′; and NLRP3, F: 5′‐CCACAAGATCGTGAGAAAACCC‐3′, R: 5′‐CGGTCCTATGTGCTCGTCA‐3′.

### 2.9 Statistical analysis

Data are presented as mean ± standard deviation (SD) and ‘n’ represents the number of independent experiments. Statistical significance was calculated using Student's two‐tailed unpaired t test or one‐way analysis of variance (ANOVA) with Holm‐Sidak's multiple comparisons test. Moreover, correlation analysis was conducted using the Pearson correlation coefficient. *P* values of <.05 were considered statistically significant. All statistical analyses were performed using the GraphPad Prism 8.0 software.

## 3 RESULTS

### 3.1 Reduced miR‐223‐3p expression up‐regulates caspase‐1 and IL‐1β in syphilis patients

**FIGURE 1:**
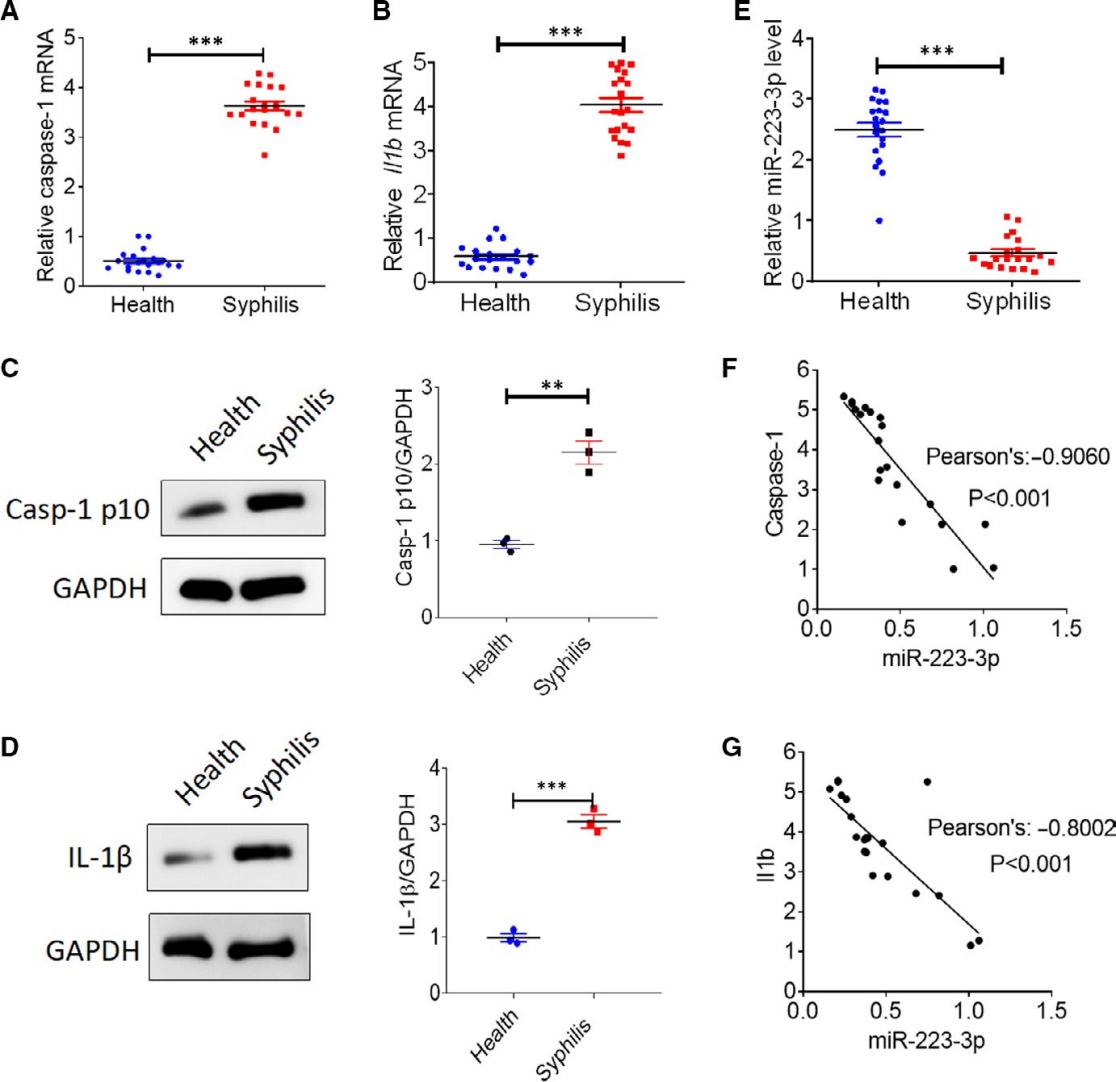
Reduced miR‐223‐3p expression is accompanied by up‐regulated caspase‐1 and IL‐1β levels in syphilis patients. Blood samples were collected from 20 syphilis patients infected with *Treponema pallidum* and 20 healthy controls. A,The mRNA expression of caspase‐1 was analysed with qRT‐PCR. B, The mRNA expression of IL‐1β was analysed with qRT‐PCR. C, The activation of caspase‐1 was analysed with immunoblotting using the anti‐Casp‐1 p10 antibody. D, The protein expression of IL‐1β was analysed with Western blotting. E, The expression of miR‐223‐3p was analysed with qRT‐PCR. F, Correlation analysis of miR‐223‐3p and caspase‐1 expression using Pearson’s correlation coefficient. G, Correlation analysis of miR‐223‐3p and IL‐1β expression using Pearson’s correlation coefficient. **P < .01 and ***P < .001 vs healthy controls, Student’s *t* test

To investigate the role of miR‐223‐3p in syphilis, we collected serum from 20 syphilis patients infected with *T pallidum* and 20 healthy controls. Similar to previous studies, caspase‐1 and IL‐1β mRNA (Figure 1A‐B) and protein (Figure 1C‐D) levels were found to be up‐regulated in syphilis patients when compared with healthy controls. Notably, we found that miR‐223‐3p levels were strongly decreased in the serum of syphilis patients (Figure 1E). Thus, we next evaluated the correlation between miR‐223‐3p expression and inflammasome activation. As shown in Figure 1F‐G, miR‐223‐3p expression levels were found to be negatively correlated with caspase‐1 and IL‐1β levels, indicating that miR‐223‐3p may play a role in the progression of syphilis.

### 3.2 Inflammasome activation and pyroptosis in rTp17‐infected HUVECs

**FIGURE 2:**
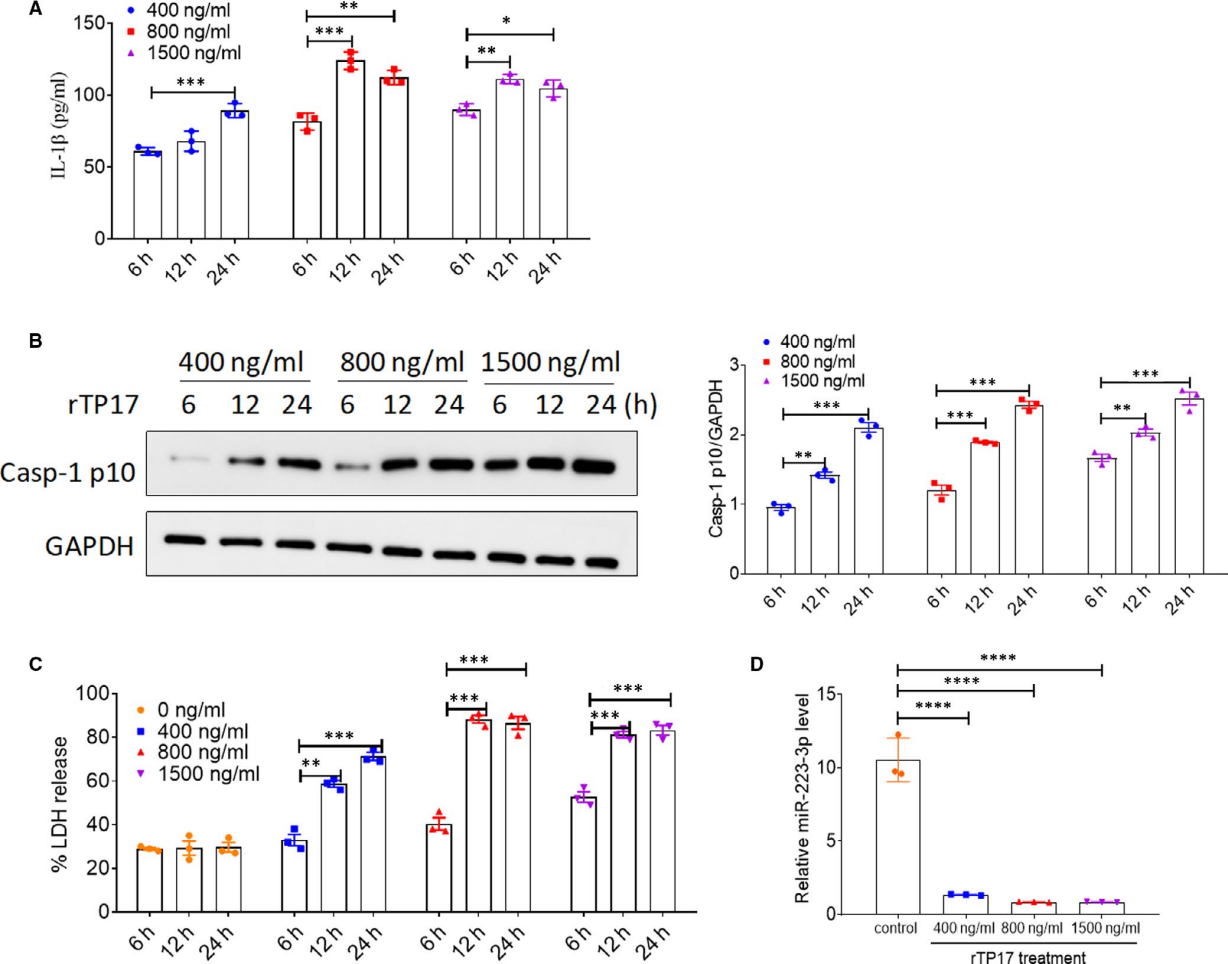
Inflammasome activation and pyroptosis occur in rTP17‐infected HUVECs. HUVECs were treated with rTp17 at various concentrations (400 ng/mL, 800 ng/mL, 1500 ng/mL) and for various time‐points (6, 12, 24 h). A, IL‐1β levels in the cell supernatants were analysed with ELISA. B, The activation of caspase‐1 in HUVECs was analysed with immunoblot using the anti‐Casp‐1 p10 antibody. C, LDH release was measured using an LDH release assay and the spontaneous release of LDH from HUVECs in the absence of rTp17 treatment was used as a control. The percentage of LDH release was calculated using the following equation: LDH release (%) = (Experimental LDH release ‐ Spontaneous LDH release) / Maximum LDH release. D, miR‐223‐3p levels in HUVECs exposed to rTp17. *P < .01, **P < .01, and ***P < .001 vs the 6‐h treatment group, Student’s *t* test

Studies on *T pallidum* membrane immunogens have provided insights into the immunopathogenesis of syphilis. Among the periplasmic lipoproteins, Tp17 has been reported to be a strong membrane immunogen and was initially recognized to play a role in inflammation during syphilis, as it reacted intensely with human syphilitic sera.^21^, ^22^ Moreover, our previous work also demonstrated that rTp17 could induce a strong inflammatory response in HUVECs (Peng et al, 2019). Therefore, in this study, we used the rTp17 as a stimulator and investigated whether the rTp17 could induce inflammasome activation and cell pyroptosis in HUVECs. As shown in Figure 2A‐D, we found that treatment with 800 ng/mL of rTp17 for 24 hours was optimal for inducing inflammasome activation and pyroptosis, as well as up‐regulated caspase‐1 activation, IL‐1β expression and LDH release. Thus, we used these conditions for all following experiments.

### 3.3 Effect of miR‐223‐3p on rTp17‐induced inflammasome activation and pyroptosis in HUVECs

**FIGURE 3:**
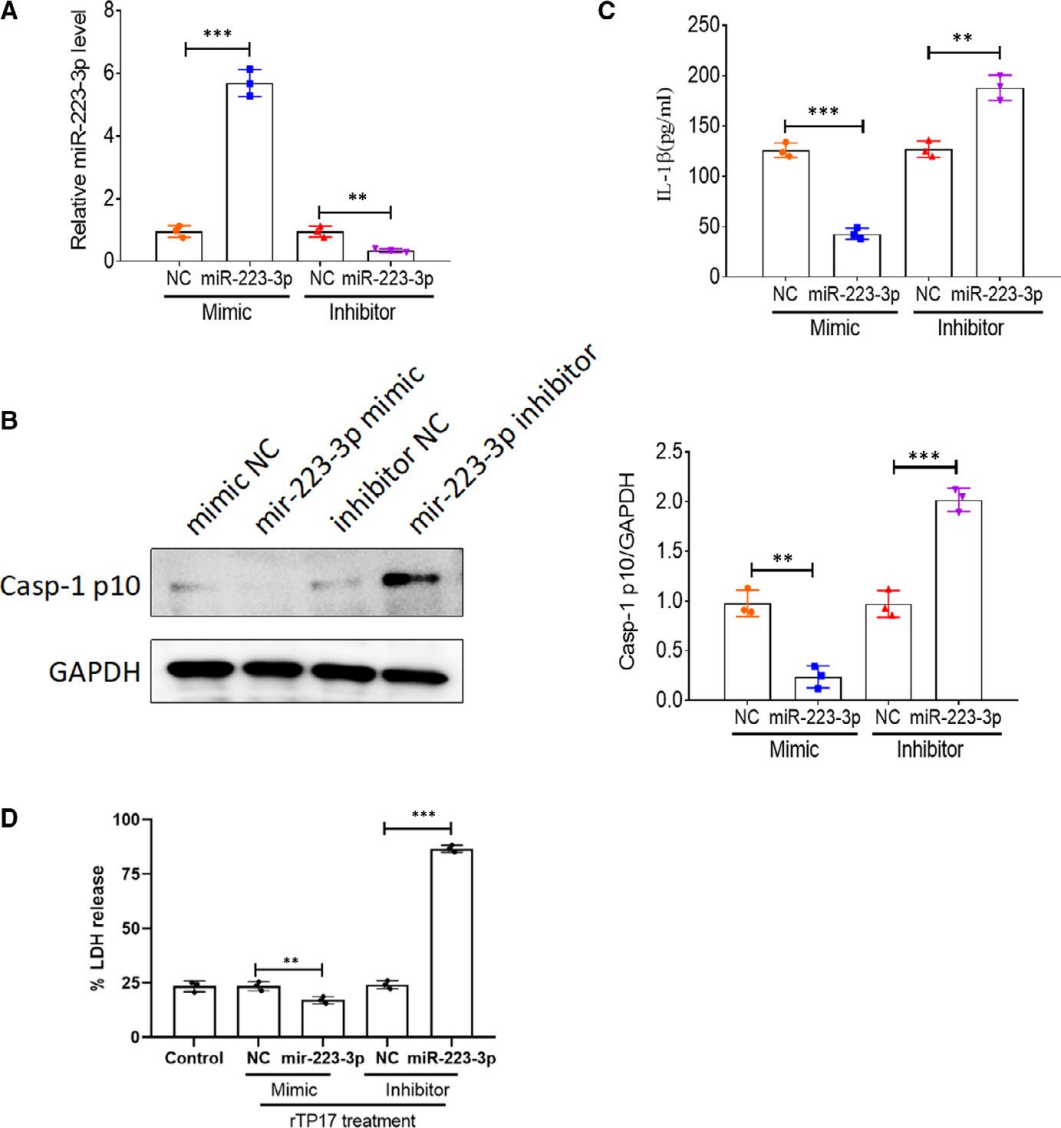
Effect of miR‐223‐3p on rTp17‐induced inflammasome activation and pyroptosis in HUVECs. HUVECs were transfected with a mimic negative control (NC), miR‐223‐3p mimic, inhibitor NC, or miR‐223‐3p inhibitor for 48 h and then the cells were treated with rTp17 at 800 ng/mL for 12 h. A, The transfection efficiency for the miR‐223‐3p mimic or inhibitor was analysed with qRT‐PCR. B, The activation of caspase‐1 in HUVECs was analysed with immunoblotting using the anti‐Casp‐1 p10 antibody. C, The protein expression of IL‐1β in HUVECs was analysed with Western blotting. D, LDH release was measured using an LDH release assay and the spontaneous release of LDH from untransfected HUVECs was used as a control. The percentage of LDH release was calculated using the following equation: LDH release (%) = (experimental LDH release ‐ spontaneous LDH release) / maximum LDH release. **P <.01 and ***P < .001 vs mimic NC or inhibitor NC or control, Student’s *t* test

To further determine the role of miR‐223‐3p in syphilis, we performed a gain‐ and loss‐of‐function experiment by transfecting the HUVECs with a miR‐223‐3p mimic, miR‐223‐3p inhibitor or negative control to assess their effect on rTp17‐mediated inflammasome activation and cell pyroptosis. We first performed qPCR to verify that the miR‐223‐3p expression level was indeed up‐regulated or down‐regulated in the HUVECs which were transfected with the miR‐223‐3p mimic or inhibitor (Figure 3A). As shown in Figure 3B‐D, the miR‐223‐3p mimic significantly decreased caspase‐1 activation and IL‐1β expression, as well as the release of LDH in rTP17‐treated HUVECs compared with the negative control. In contrast, transfection with the miR‐223‐3p inhibitor significantly increased inflammasome activation and cell pyroptosis (Figure 3B‐D). Taken together, our results demonstrate that miR‐223‐3p plays a role in the inhibition of inflammasome activation and cell pyroptosis.

### 3.4 NLRP3 is the downstream target of miR‐223‐3p in HUVECs

**FIGURE 4:**
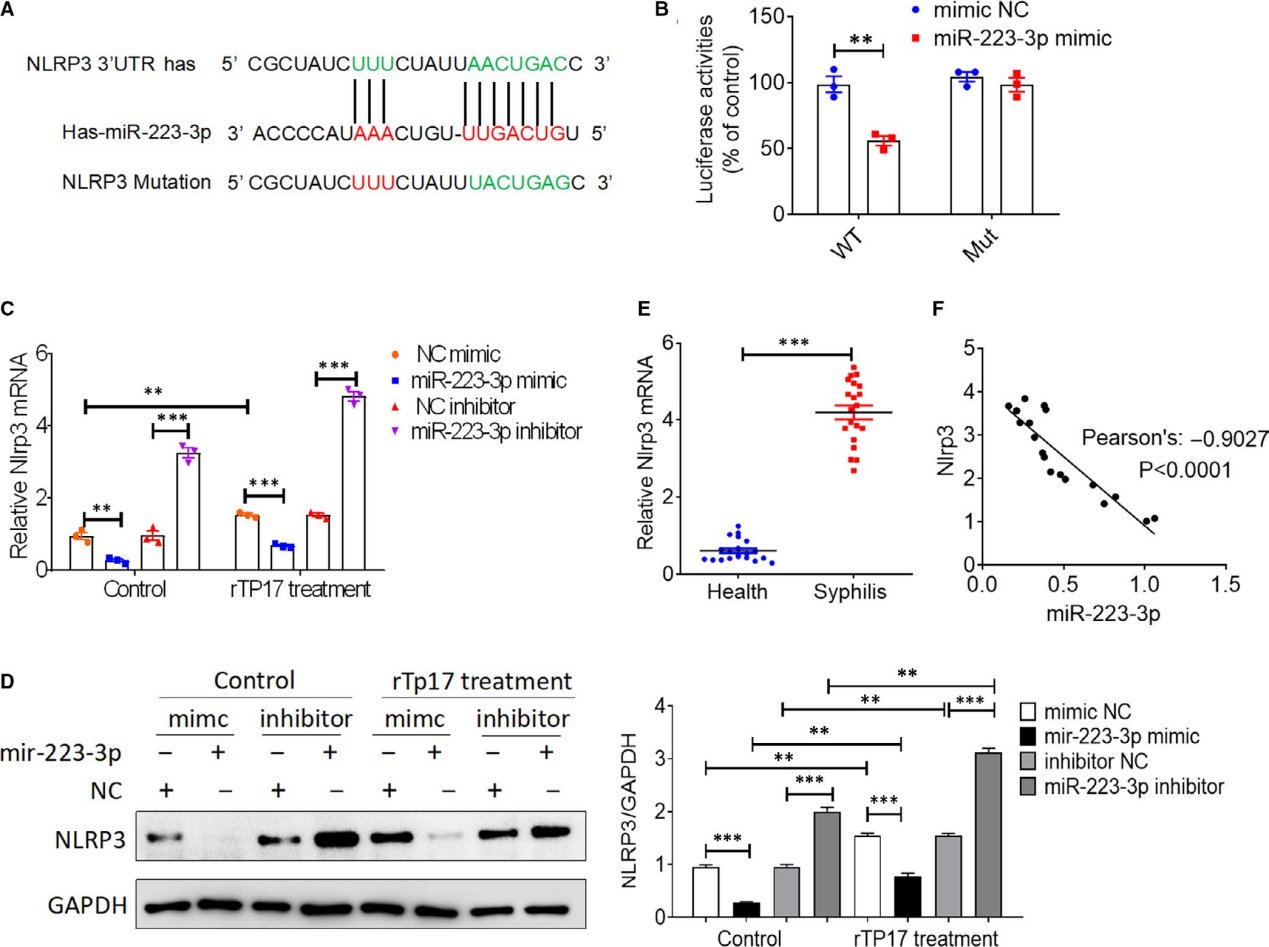
NLRP3 is the downstream target of miR‐223‐3p in HUVECs. A, Alignment of miR‐223‐3p and its corresponding complementary binding sequence in the 3ʹ‐UTR of NLRP3 using the TargetScan bioinformatics algorithm. B, HUVECs were transfected with luciferase reporter plasmids containing the wild‐type (WT) or mutant (Mut) NLRP3 3ʹ‐UTR or an empty vector (Vector) together with a miR‐223‐3p mimic or mimic negative control (NC) and luciferase activity was measured at 48 h after transfection. C‐D, HUVECs were transfected with a mimic NC, miR‐223‐3p mimic, inhibitor NC or miR‐223‐3p inhibitor for 48 h, and then, the cells were treated with or without rTp17 at 800 ng/mL for 12 h. NLRP3 expression was analysed with qRT‐PCR and Western blotting. E, NLRP3 expression was analysed with qRT‐PCR in blood samples from syphilis patients infected with *T. pallidum* and healthy controls (n=20 biological replicates). F, Correlation analysis of miR‐223‐3p and NLRP3 expression using Pearson’s correlation coefficient. **P < .01 and ***P < .001 vs healthy controls, Student’s *t* test

To explore the mechanism by which miR‐223‐3p regulates inflammasome activation and cell pyroptosis in HUVECs, we investigated the downstream target genes associated with inflammasome activation. It was reported that NLRP3 transcription is tightly regulated by miR‐223‐3p through an evolutionarily conserved binding interaction between the 3′‐UTR of the NLRP3 mRNA and miR‐223‐3p in macrophages and hepatocellular carcinoma (HCC) cells. To confirm whether miR‐223‐3p could regulate NLRP3 expression in rTp17‐treated HUVECs, we first constructed the 3′‐UTR of NLRP3 and inserted a point mutation into the site to prevent miR‐223‐3p binding (Figure 4A). As shown in Figure 4B, co‐transfection with miR‐223‐3p reduced the luciferase activity of HUVECs transfected with the NLRP3 containing the wild‐type 3′‐UTR (NLRP3‐3′‐UTR^WT^) but not that of HUVECs transfected with the NLRP3 containing the mutated 3ʹ‐UTR (NLRP3‐3′‐UTR^Mut^). Next, we further assessed the correlation between NLRP3 and miR‐223‐3p expression. Interestingly, NLRP3 expression in HUVECs was notably inhibited at the mRNA and protein levels following treatment with a miR‐223‐3p mimic in either the presence or absence of an rTp17 infection. In contrast, treatment with the miR‐223‐3p inhibitor was shown to up‐regulate NLRP3 expression (Figure 4C‐D). Moreover, an increase in NLRP3 expression was also observed in syphilis patients when compared to healthy control, which was negatively correlated with the expression of miR‐223‐3p (Figure 4E‐F). These data suggest that miR‐223‐3p could target NLRP3 in order to down‐regulate its expression in HUVECs infected with rTp17.

### 3.5 NLRP3 rescues the effect of miR‐223‐3p on rTp17‐induced inflammasome activation and cell pyroptosis

**FIGURE 5:**
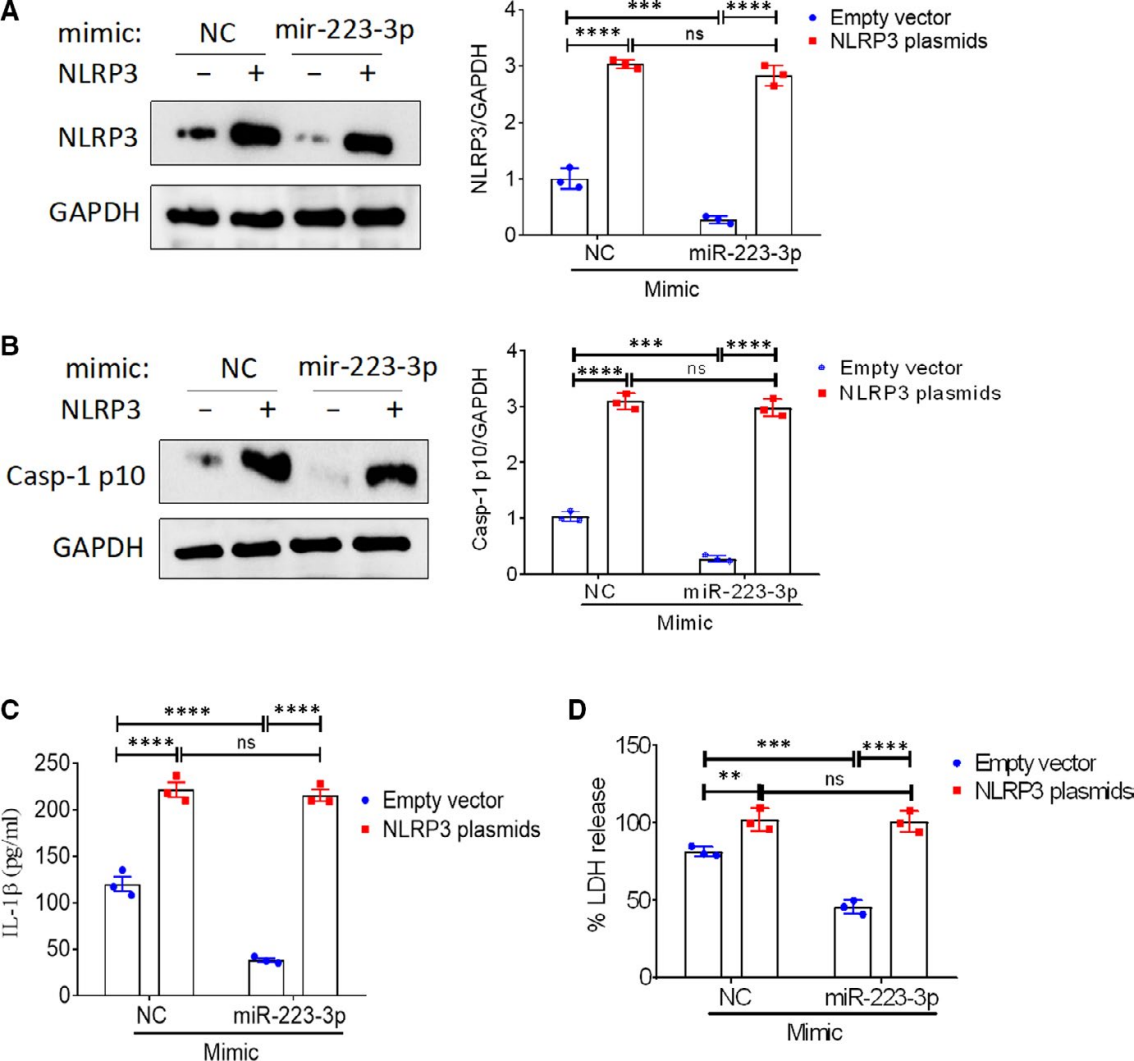
NLRP3 overexpression rescues the effect of miR‐223‐3p on rTp17‐induced inflammasome activation and cell pyroptosis. HUVECs transfected with an empty vector or an NLRP3 plasmid were further transfected with or without a mimic negative control (NC) or miR‐223‐3p mimic for 48 h. A, The expression of NLRP3 in HUVECs was analysed with Western blotting. The transfected cells were then treated with rTp17 at 800 ng/mL for 12 h. B, The activation of caspase‐1 in HUVECs was analysed with immunoblotting using an anti‐Casp‐1 p10 antibody. C, The protein expression of IL‐1β in HUVECs was analysed with Western blotting. D, LDH release was measured using an LDH release assay and the spontaneous release of LDH from HUVECs transfected with an empty vector + mimic NC was used as a control. The percentage of LDH release was calculated using the following equation: LDH release (%) = (experimental LDH release ‐ spontaneous LDH release) /maximum LDH release. **P < .01 and ***P < .001 vs mimic NC or inhibitor NC or control, Student’s *t* test

**FIGURE 6:**
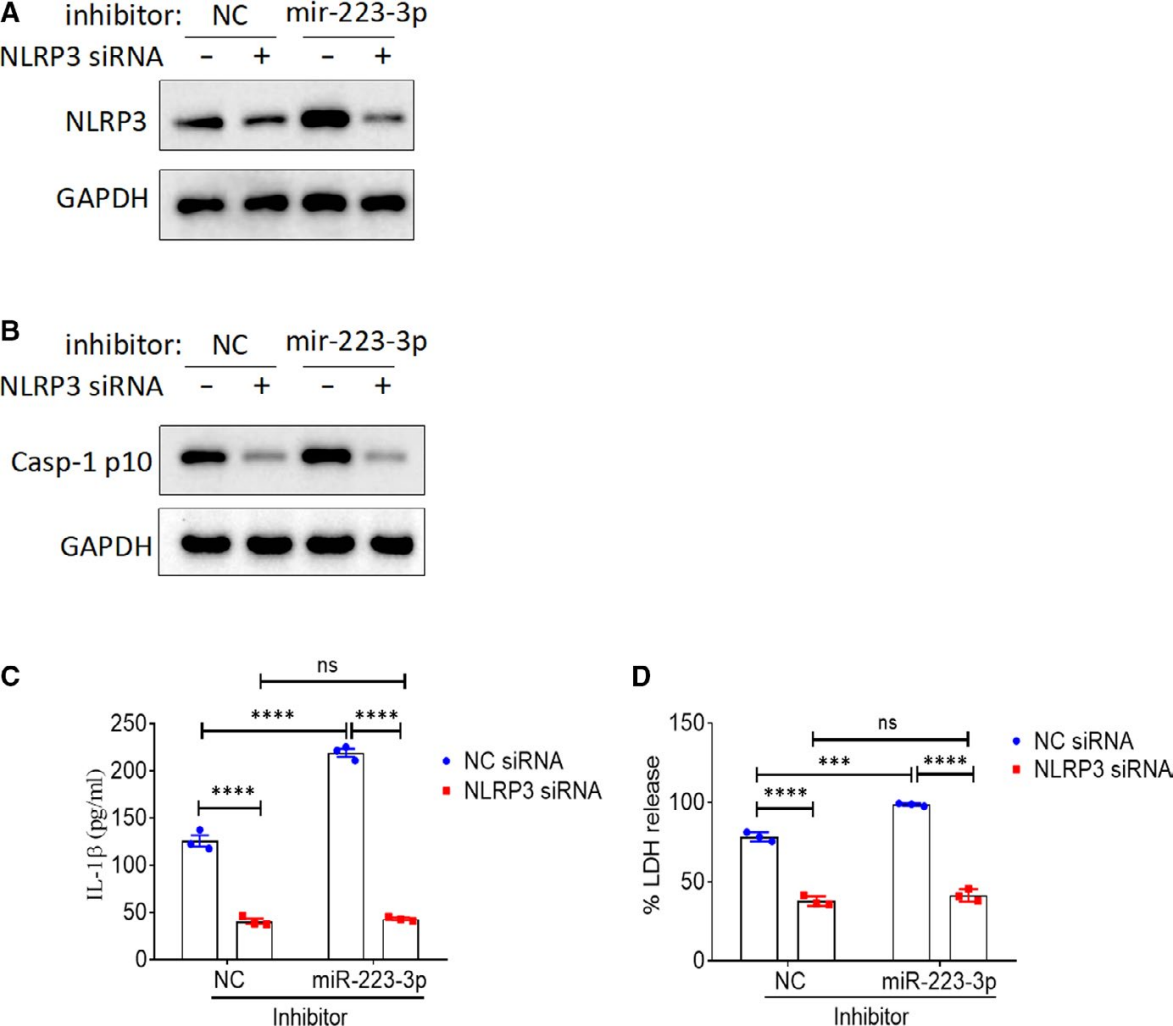
Effect of NLRP3 knockdown on the miR‐223‐3p‐mediated regulation of inflammasome activation and cell pyroptosis. HUVECs transfected with a negative control (NC) or NLRP3 siRNA were further transfected with or without an inhibitor NC or miR‐223‐3p inhibitor for 48 h. A, The expression of NLRP3 in HUVECs was analysed with Western blotting. The transfected cells were then further treated with rTp17 at 800 ng/mL for 12 h. B, The activation of caspase‐1 in HUVECs was analysed with immunoblotting using an anti‐Casp‐1 p10 antibody. C, The protein expression of IL‐1β in HUVECs was analysed with Western blotting. D, LDH release was measured. The percentage of LDH release was calculated using the following equation: LDH release (%) = (experimental LDH release ‐ spontaneous LDH release) / maximum LDH release. **P < .01 and ***P < .001 vs mimic NC or inhibitor NC or control, Student’s *t* test

To further confirm that the miR‐223‐3p‐mediated inhibitory effect on inflammasome activation and cell pyroptosis was dependent on its target NLRP3, we constructed an NLRP3 plasmid to perform the rescue experiment. The immunoblot results showed that miR‐223‐3p‐mediated down‐regulation of NLRP3 was completely rescued by NLRP3 overexpression (Figure 5A). We then evaluated the effects of NLRP3 overexpression on miR‐223‐3p‐mediated inflammasome activation and cell pyroptosis. In accordance with previous studies, treatment with a miR‐223‐3p mimic or inhibitor dramatically decreased or increased rTp17‐induced inflammasome activation, cell pyroptosis, caspase‐1 activation, IL‐1β secretion, and LDH release. Additionally, NLRP3 overexpression significantly rescued cells from this suppressive effect (Figure 5B‐D). In order to determine a real link between miR‐223‐3p and NLRP3, we knocked down NLRP3 expression using a specific siRNA. Western blot analysis revealed that miR‐223‐3p inhibitor‐induced up‐regulation of NLRP3 was abolished by NLRP3 knockdown (Figure 6A). Accordingly, knockdown of NLRP3 obviously rescued cells from miR‐223‐3p‐mediated inflammasome activation and cell pyroptosis (Figure 6B‐D). Taken together, our results demonstrate that in endothelial cells, miR‐223‐3p‐mediated suppression of rTp17‐induced inflammasome activation and cell pyroptosis is largely dependent on NLRP3 targeting.

## 4 DISCUSSION

In this study, we first demonstrated that miR‐223‐3p expression levels were decreased in syphilis patients when compared to healthy control, which was negatively correlated with caspase‐1 activation and IL‐1β expression. Furthermore, we found that treatment with a miR‐223‐3p mimic strongly suppressed *T pallidum*‐induced caspase‐1 activation and IL‐1β production, as well as LDH release, by directly targeting NLRP3. Conversely, treatment with a miR‐223‐3p inhibitor was found to have the opposite effect. Notably, we showed that NLRP3 overexpression could block the effect of miR‐223‐3p on *T pallidum*‐induced inflammasome activation and pyroptosis. Thus, our study provides novel insights with regard to the role of miR‐223‐3p in *T pallidum*‐mediated inflammasome activation and pyroptosis and suggests that miR‐223‐3p might be a promising therapeutic target for syphilis.

Syphilis is a chronic infectious disease caused by the spirochaete *T pallidum* and is usually venereal in origin but often congenital. Accumulating evidence has shown that *T pallidum* infection‐induced tissue inflammation and injury, including inflammasome activation, contributes to the development of syphilis.^23^, ^24^ Moreover, a recent work revealed that the outer membrane protein Tp92 of *T pallidum* could induce caspase‐1‐dependent pyroptosis in HUVECs.^11^ In addition, our previous study also demonstrated that Tp17, which is a strong membrane immunogen and reacts intensely with human syphilitic sera, induced TLR4 pathway activation and a proinflammatory response in HUVECs.^14^ However, whether Tp17 could induce inflammasome activation and caspase‐1‐dependent pyroptosis remained unclear. Here, we found that rTP17 stimulation could lead to caspase‐1 activation and LDH release in the HUVECs.

Currently, miRNAs have emerged as an important therapeutic target for many diseases, including metabolic diseases, inflammation and cancer. MiR‐223‐3p was found to be down‐regulated in several tumour types and is known to be involved in regulating cell growth and apoptosis.^25^, ^26^ Furthermore, previous studies have shown that miR‐223‐3p has the ability to dampen the inflammatory response.^27^, ^28^ However, its role in syphilis is poorly understood. In this study, we found that miR‐223‐3p expression was significantly down‐regulated in human syphilis tissues when compared to healthy controls. Moreover, using HUVECs with a miR‐223‐3p mimic or inhibitor, we demonstrated that miR‐223‐3p up‐regulation inhibited the rTP17‐induced caspase‐1 activation and IL‐1β production, as well as LDH release. Conversely, miR‐223‐3p down‐regulation strongly promoted caspase‐1 activation, IL‐1β production and LDH release. Thus, we speculated that miR‐223‐3p might play a protective role against *T pallidum* infection. Additionally, exosomes, which are a type of secreted vesicle that range in diameter from 30 to 100 nm and are considered mediators of intercellular communication through their cargo, were reported to play a role in the therapeutic effects of miRNAs in several diseases.^14^, ^29^, ^30^, ^31^ Our previous study also showed that miR‐216a‐5p‐containing exosomes suppressed rTp17‐induced inflammatory responses. Thus, it would be interesting to investigate whether exosomes containing miR‐223‐3p and miR‐216a‐5p have a synergistic effect and exert a stronger inhibition of *T pallidum*‐induced inflammatory injury.

Recently, several reports have confirmed that miR‐223 directly targets NLRP3.^32^, ^33^, ^34^ Our results also confirmed that miR‐223‐3p negatively regulates the NLRP3 expression, by using a dual‐luciferase assay, and also that this process occurs in syphilis patients and HUVECs stimulated with rTP17. Moreover, we found that NLRP3 overexpression could block the inhibitory effects of miR‐223‐3p on inflammasome activation and pyroptosis in the HUVECs stimulated with rTP17.

In conclusion, we found that miR‐223‐3p was significantly down‐regulated in syphilis patients when compared to healthy controls, whereas NLRP3 and caspase‐1 expression were dramatically up‐regulated in syphilis patients. Moreover, miR‐223‐3p inhibited *T pallidum*‐induced caspase‐1 activation, IL‐1β production and LDH release in HUVECs. Mechanistically, gain‐ and loss‐of‐function experiments demonstrated that miR‐223‐3p targets NLRP3 in order to suppress inflammasome activation and pyroptosis, highlighting the potential of miR‐223‐3p as a therapeutic target for the clinical treatment of syphilis.

## 5 AUTHOR STATEMENT

Syphilis, which is caused by *Treponema pallidum* subsp *pallidum* (*T pallidum*) infection, continues to be a prevalent disease globally, involving severe tissue inflammation. Recently, accumulating evidence has illustrated the vital role of inflammasome activation and pyroptosis in the development of syphilis. An increasing number of studies have reported that microRNAs (miRNAs) are dysregulated in syphilis patients. We found that miR‐223‐3p, which was reported to be involved in the regulation of inflammasome activation and cancer development via NLRP3 targeting, was greatly down‐regulated in syphilis patients. However, the mechanism by which miR‐223‐3p regulates inflammasome activation and pyroptosis in HUVECs, and whether that then further leads to syphilis development remain unknown. This work contributes to our understanding of syphilis pathogenesis and also supplies a potential therapeutic target for syphilis.

## CONFLICT OF INTEREST

The authors confirm that there are no conflicts of interest.

## AUTHOR CONTRIBUTIONS

**Fu‐quan Long:** Conceptualization (lead); Funding acquisition (equal); Investigation (equal); Project administration (lead); Resources (equal); Writing‐original draft (equal); Writing‐review & editing (equal). **Cai‐xia Kou:** Formal analysis (equal); Investigation (equal). **Ke Li:** Supervision (equal); Validation (equal); Visualization (equal). **Juan Wu:** Investigation (equal); Methodology (equal); Project administration (equal). **Qian‐qiu Wang:** Conceptualization (equal); Data curation (lead); Funding acquisition (equal); Methodology (lead); Project administration (equal); Supervision (lead); Visualization (equal).

## Data Availability

The data that support the findings of this study are available from the corresponding author upon reasonable request.
